# Association between Recreational Screen Time and Sleep Quality among Adolescents during the Third Wave of the COVID-19 Pandemic in Canada

**DOI:** 10.3390/ijerph19159019

**Published:** 2022-07-25

**Authors:** Lydi-Anne Vézina-Im, Dominique Beaulieu, Stéphane Turcotte, Joanie Roussel-Ouellet, Valérie Labbé, Danielle Bouchard

**Affiliations:** 1 Département des Sciences de la Santé, Université du Québec à Rimouski (UQAR), Campus de Lévis, 1595 Boulevard Alphonse-Desjardins, Lévis, QC G6V 0A6, Canada; dominique_beaulieu@uqar.ca (D.B.); joanie.roussel-ouellet@uqar.ca (J.R.-O.); 2 Centre de Recherche du CISSS de Chaudière-Appalaches, 143 rue Wolfe, Lévis, QC G6V 3Z1, Canada; stephane_turcotte@ssss.gouv.qc.ca; 3 Axe Santé des Populations et Pratiques Optimales en Santé, Centre de Recherche du CHU de Québec, 2400 Avenue D’Estimauville, Quebec, QC G1E 6W2, Canada; 4 CHAU-Hôtel-Dieu de Lévis, 143 Rue Wolfe, Lévis, QC G6V 3Z1, Canada; v_labbe2000@yahoo.ca; 5 Laboratoire du Sommeil, Hôtel-Dieu de Lévis, 143 Rue Wolfe, Lévis, QC G6V 3Z1, Canada; danielle.bouchard.cisssca@ssss.gouv.qc.ca

**Keywords:** sleep quality, screen time, concurrent screen use, adolescent, COVID-19

## Abstract

The study objective was to verify whether recreational screen time was associated with sleep quality among adolescents during the third wave of the COVID-19 pandemic in Canada. Data collection took place in four high schools in the region of Chaudière-Appalaches (Quebec, Canada) from the end of April to mid-May 2021. Recreational screen time and sleep quality were measured using the French versions of validated questionnaires specifically designed for adolescents. A total of 258 adolescents (14–18 years; 66.3% girls) answered the online survey. Adolescent boys had a higher total mean recreational screen time (454.3 ± 197.5 vs. 300.5 ± 129.3 min/day, *p* < 0.0001) and a higher total mean sleep quality score (4.2 ± 0.9 vs. 3.9 ± 0.8, *p* = 0.0364) compared to girls. Recreational screen time (β = −0.0012, *p* = 0.0005) and frequency of concurrent screen use (sometimes: β = −0.3141, *p* = 0.0269; often: β = −0.4147, *p* = 0.0048; almost always or always: β = −0.6155, *p* = 0.0002) were negatively associated with sleep quality while being a boy (β = 0.4276, *p* = 0.0004) was positively associated with sleep quality and age (*p* = 0.6321) was not. This model explained 16% of the variance in adolescents’ sleep quality. Public health interventions during and after the COVID-19 pandemic should target recreational screen time, concurrent screen use and especially girls to possibly improve sleep quality and promote adolescents’ physical and mental health.

## 1. Introduction

Screens are increasingly present in our society, and most adolescents have access to different screens (e.g., television, smart phone, computer, tablet, or gaming consoles) at home. The Canadian 24 h movement guidelines [1] recommend children and adolescents (5–17 years) limit recreational screen time to a maximum of 2 h/day. However, only 53.3% of Canadian children and adolescents (5–17 years) adhered to this public health recommendation, and the average recreational screen time in this population was of 3.8 h/day in 2018–2019 [2]. During the peak of the COVID-19 pandemic, people in several countries (including Canada) were confined at home, and this includes adolescents who had remote school. Adolescents increased their recreational screen time during the peak of the COVID-19 pandemic likely because they were more often at home. In fact, 93.4% of adolescents (12–17 years) had a recreational screen time of over 2 h/day during the first wave (April 2020) [3] and 83.5% during the second wave (October 2020) [4] of the COVID-19 pandemic in Canada.

Excessive screen time can have many negative consequences in adolescents, such as adverse impacts on cognitive and psychosocial development (e.g., attention and learning difficulties) [5], increase risk behaviors (e.g., drug and alcohol consumption) [6], influence other lifestyle habits (e.g., junk food and sugar-sweetened beverage consumption in front of screens) [7], and contribute to health issues, such as overweight, depression and sleep problems [8]. Short and poor sleep quality is also associated with health issues among adolescents, such as obesity [9] and mental health problems (e.g., depression and anxiety) [10]. The COVID-19 pandemic affected Canadian adolescents’ sleep. Adolescents (12–17 years) increased their sleep duration during the first wave (April 2020), with 72.2% of adolescent adhering to the Canadian 24 h movement guidelines of sleeping 9–11 h/night for children and adolescents aged 5–13 years and 8–10 h/night for those aged 14–17 years [3]. However, by the second wave (October 2020) of the COVID-19 pandemic in Canada, their sleep duration had decreased, with only 59.5% adhering to these sleep recommendations [4]. Sleep duration went from 9.0 h/night during the first wave to 8.3 h/night during the second wave of the COVID-19 pandemic in Canada [4]. Adolescents (12–17 years) from the province of Quebec (Canada) reported an improvement in their sleep quality in June 2020, near the end of the first wave of the COVID-19 pandemic that ended in July 2020 in the province of Quebec [11]. Another study among adolescents (12–17 years) from the province of Quebec reported that sleep quality significantly differed between winter, spring, summer, and autumn 2020, with a higher proportion of adolescents reporting good sleep quality during summer (July and August) compared to spring (March and June) 2020 [12].

Recent data suggest that different lifestyle habits, such as diet, physical activity, sedentary behavior and sleep, are all related in adolescents [13]. Adolescents with high levels of sedentary behavior, such as excessive recreational screen time, are more likely to have inadequate and poor sleep quality, ultimately increasing their likelihood of having obesity [14]. This could be because excessive screen time leaves less time for physical activity and sleep in a day. The Canadian 24 h movement guidelines for children and adolescents use this reasoning and integrate physical activity, sedentary behavior (including recreational screen time), and sleep recommendations [1]. For example, it is recommended that children and adolescents replace recreational screen time by more moderate to vigorous physical activity and preserve enough time for sleep at night [1]. Links between lifestyle habits can be complex. For instance, there is conflicting information about whether moderate to vigorous physical activity can improve sleep in adolescents, with some studies reporting a positive association [15] and others finding no association between both [16], and it is unclear which timing (i.e., morning, afternoon, or evening) of physical activity is associated with better sleep [17]. Lack of sleep can also be associated with higher consumption of calories in adolescents [18,19]. More research is therefore needed to understand how different lifestyle habits can be linked together and influence risk of overweight and obesity in children and adolescents [13], in order to have a holistic approach to obesity prevention in adolescents [20].

Some behaviors are considered healthy sleep habits as they can promote adequate sleep. For instance, it is recommended that adolescents avoid any screen time within 1 hour of bedtime and the presence of electronic devices in the bedroom [21] since both daytime and screen time within 1 hour of bedtime can negatively affect sleep [22]. In a systematic review, 90% of the studies reported that screen time negatively affected adolescents’ sleep (e.g., shorter sleep duration and later bedtime) [23]. Another systematic review reported that screen time in adolescents (13–15 years) was associated with problems falling asleep and poor sleep quality [24]. Using many screens simultaneously (i.e., concurrent screen use) has also been associated with shorter sleep duration in adolescents (11–14 years) [25]. Unfortunately, many adolescents use screens within 1 hour of bedtime and have concurrent screen use as well according to pre-pandemic data. In a study among adolescents (16–19 years) in Norway, over 80% of adolescents reported using their computers and cell phones within 1 hour of bedtime [22] while in a study among adolescent girls (11–14 years) in the United Kingdom, 65% and 36% of them had concurrent screen use in the evening and before bedtime, respectively [25].

Recreational screen time can vary between adolescent boys and girls according to pre-pandemic data. A lower proportion (7.1%) of Canadian boys (10–17 years) adhered to the Canadian 24 h movement guideline of a maximum of 2 h/day of recreational screen time compared to girls (8.9%) in 2013–2014 [26]. Another study conducted in 2013–2014 among Canadian adolescents (13–18 years) also reported that boys were less likely to adhere to this recommendation compared to girls [27]. Canadian adolescent boys (12–17 years) reported playing more video and computer games compared to girls in data from multiple national cross-sectional surveys in Canada [28]. Similarly, adolescent boys in Norway mentioned using their gaming consoles more compared to girls who primarily used their cell phones and MP3 players within 1 hour of bedtime [22]. Biological sex could also affect adolescents’ sleep quality according to pre-pandemic data. Adolescent girls (15–17 years) were more likely to have poor sleep quality [29]. Adolescent girls (12–16 years) also reported greater insomnia symptom severity, longer wakefulness at night, lower sleep duration, and lower sleep efficiency (i.e., ratio of time spent asleep over total time spent in bed) compared to boys [30]. Age is another variable that could affect sleep in adolescents according to pre-pandemic data. Older adolescents had a shorter sleep duration and went to bed later on school days compared to younger adolescents [31]. In adolescent girls, higher sexual maturity (i.e., later Tanner stage) was associated with increased odds and severity of insomnia (e.g., difficulty initiating and/or maintaining sleep and/or early morning awakenings) compared to boys [32].

The COVID-19 pandemic contributed to increase adolescents’ recreational screen time, and this may have also affected their sleep quality. Given that previous work has shown an association between the two, increases in recreational screen time were likely associated with declines in sleep quality during the peak of the COVID-19 pandemic. It is important to determine whether public health interventions should suggest limiting recreational screen time as a way to potentially improve adolescents’ sleep quality during and after the COVID-19 pandemic. The objective of this study was to verify if recreational screen time was associated with sleep quality among adolescents during the third wave of the COVID-19 pandemic in Canada.

## 2. Materials and Methods

This investigation is part of a larger study on the psychosocial correlates of recreational screen time based on the Reasoned Action Approach [33]. Inclusion criteria were: (1) being between 14–18 years of age and (2) attending a high school in the region of Chaudière-Appalaches (Quebec, Canada). The project was approved by the Research Ethics Committee of the Centre intégré de santé et de services sociaux (CISSS) de Chaudière-Appalaches (2021-853). Prior to the main study, a two-week test–retest study among 38 adolescents (14–15 years) was conducted in December 2020 to verify the psychometric qualities of the questionnaires measuring adolescents’ recreational screen time and sleep quality. These 38 adolescents were from a different high school than the ones selected for the main study. Data collection for the main study took place in 12 classes of four high schools (three classes per school: one in 3rd year, one in 4th year and one in 5th year of high school) from late April to mid-May 2021 (i.e., during the third wave of the COVID-19 pandemic in Canada). Questionnaires were completed online during class. Three CAD$ 25 gift cards for a local sports store were drawn among each class for a total of 36 gift cards. Written informed consent to participate in the study was obtained from each participant.

Recreational screen time was measured using the validated Screen Time-Based Sedentary Behavior Questionnaire [34]. This questionnaire had acceptable test–retest reliability with almost all κ-values > 0.70 among 183 adolescents (12–18 years) and it was also validated with objectively measured sedentary time among 2048 adolescents of the same age [34]. It contains 12 questions that measure television viewing, computer games, console games, Internet for study (educational) and non-study (recreational) reasons, and studying during week and weekend days. Our version contained eight questions as the items on time spent studying and using Internet for study reasons were not included. Only the question on Internet use for non-study reasons (e.g., chatting, YouTube, Netflix) was included since (1) we were interested in recreational screen time; (2) many high school students had remote school during the peak of the COVID-19 pandemic in Canada; and (3) some high schools include electronic devices (e.g., tablets) in their programs. Prior to the items of the Screen Time-Based Sedentary Behavior Questionnaire, adolescents were asked to estimate in hours their weekly recreational screen time to verify if measuring time spent using different screens separately could overestimate recreational screen time. A question on frequency of concurrent screen use (e.g., watching television and using a smart phone at the same time) was added after those of the Screen Time-Based Sedentary Behavior Questionnaire to assess whether this is common in adolescents and if it can affect their sleep quality.

Sleep quality was measured using the validated short version of the Adolescent Sleep–Wake Scale (ASWS short version) [35,36]. This questionnaire had acceptable internal consistency with all Cronbach alpha coefficients > 0.70 among a sample of 491 adolescents (12–18 years) without and with pediatric health conditions (i.e., non-disease-related chronic pain, sickle cell disease, traumatic brain injury, or depressive disorders) [35]. It also had all Cronbach alpha coefficients > 0.70 among a sample of 467 adolescents (12–18 years) from an economically disadvantage community, except for the going to bed subscale [36]. The ASWS short version contains 10 items that measure four dimensions of adolescents’ sleep quality: going to bed, falling asleep, reinitiating sleep, and returning to wakefulness. In previous versions of the ASWS short version [35,36], the subscales on falling asleep and reinitiating sleep were combined, while in the original version of the ASWS that contained 28 items, they were considered separately [37]. Knowing if adolescents have more trouble falling asleep or reinitiating sleep after waking up at night could be relevant for public health interventions. Therefore, the results on falling asleep and reinitiating sleep will be reported both separately and combined. French versions translated by a certified translator of both questionnaires were used given that our population was French speaking. Sociodemographic data (age, biological sex, gender and school level) were also collected to describe the sample and add those variables as potential covariates in the statistical analyses.

### Statistical Analyses

Time spent using each screen was added and divided by seven to obtain a total recreational screen time per day [34]. A mean score of sleep quality was computed (possible range: 1–6), with higher scores indicating better sleep quality [35,36]. The temporal stability of the French versions of the Screen Time-Based Sedentary Behavior Questionnaire and the ASWS short version was verified in the test–retest study by computing intra-class correlations (ICC) [38] with their 95% confidence intervals (CI). The criteria of Fermanian [39] were used to classify ICC whereby ICC between 0 and 0.30 are very bad, 0.31 to 0.50 are mediocre, 0.51 to 0.70 are moderate, 0.71 to 0.90 are good, and > 0.91 are very good. Cronbach alpha coefficients (α) [40] were computed to verify the internal consistency of the French version of the ASWS short version and each of its subscales in the main study. Cronbach alpha coefficients were classified according to the criteria of Nunnally [41] whereby α > 0.70 are considered adequate. No Cronbach alpha coefficient was calculated for the French version of the Screen Time-Based Sedentary Behavior Questionnaire, since this instrument measures time spent using different screens which might not be correlated. Descriptive statistics, including means, standard deviations, medians, interquartile ranges and percentages, were computed. Significant differences in recreational screen time and sleep quality between adolescent girls and boys were identified using independent *t*-test, Mann–Whitney U test, chi-squared, and Fisher’s exact test analyses.

A calculation was performed to verify if the sample size was sufficient to perform linear regression analyses. According to recommendations for the ratio of number of cases to independent variables for multiple regression analyses [42], a minimum of 50 + 8 (number of predictors) is needed for testing a regression model and a minimum of 104 + number of predictors is needed for testing individual predictors. The present study had four predictors of sleep quality (i.e., recreational screen time, frequency of concurrent screen use, biological sex, and age), this gives 50 + 8 (4) = 82 and 104 + 4 = 108. Our final sample of 258 adolescents is thus sufficient to perform linear regression analyses.

Linear regression analyses and its percentage of variance explained (R^2^) were computed to determine if recreational screen time, frequency of concurrent screen use, biological sex, and age were associated with adolescents’ sleep quality. Moderation analyses were also conducted to determine if some variables that were significantly associated with adolescents’ sleep quality interacted with each other (recreational screen time × concurrent screen use, recreational screen time × biological sex, and concurrent screen use × biological sex). An independent t-test analysis was used to determine if adhering to the Canadian 24 h movement guideline of a maximum of 2 h/day of recreational screen time was associated with better sleep quality. The alpha level was set at *p* < 0.05, and all analyses were performed in SAS version 9.4 (SAS Institute, Cary, NC, USA).

## 3. Results

### 3.1. Test–Retest Study

The French versions of the Screen Time-Based Sedentary Behavior Questionnaire (television: ICC = 0.80, 95% CI = 0.61–0.89; computer games: ICC = 0.65, 95% CI = 0.33–0.82; console games: ICC = 0.85, 95% CI = 0.71–0.92; Internet for non-study reasons: ICC = 0.77, 95% CI = 0.57–0.88) and the ASWS short version (ICC = 0.90, 95% CI = 0.80–0.95) had moderate to good temporal stability according to the criteria of Fermanian [39].

### 3.2. Main Study

A total of 271 adolescents answered the online survey on recreational screen time and on sleep quality. A total of 258 participants (95.2%) were kept in the statistical analyses as seven participants had given unlikely answers and six participants had completed the questionnaires in less than 8 min and also given unlikely answers. Average time to complete the online survey was 15 min. A total of 69.8% of adolescents were 15–16 years of age, 66.3% were of female sex (biological sex) and 64.3% identified as girls (gender) (see Table 1). Given that genetic or hormonal factors can influence sleep quality and since biological sex had no missing data compared to gender, biological sex was used in all the statistical analyses looking at sex differences. Adolescent boys and girls were of similar age (15.5 ± 1.1 vs. 15.6 ± 0.9 years, *p* = 0.5308). The French version of the ASWS short version (α = 0.82) and each of its subscale (going to bed: α = 0.83, falling asleep: α = 0.72, reinitiating sleep: α = 0.77, returning to wakefulness: α = 0.85) had adequate internal consistency according to the criteria of Nunnally [41]. Results were similar when the subscales on falling asleep and reinitiating sleep were combined (α = 0.76).

There were some significant differences between adolescent girls and boys in terms of recreational screen time (see Table 2). Boys had a higher total recreational screen time compared to girls (*p* < 0.0001). Girls reported a mean of 300.5 min/day (approximately 5 h/day) of total recreational screen time while boys reported a mean of 454.3 min/day (approximately 7 h and 34 min/day) of total recreational screen time. The sources of recreational screen time also differed between adolescent girls and boys. Boys mentioned more time playing console and computer games (*p* < 0.0001) compared to girls. The main sources of recreational screen time in girls were, in decreasing order: Internet for non-study reasons (e.g., chatting, YouTube, Netflix) (mean = 185.1 min/day, approximately 3 h and 5 min/day), television viewing (mean = 73.2 min/day, approximately 1 hour and 13 min/day), console games (mean = 24.7 min/day) and computer games (mean = 17.6 min/day). The main sources of recreational screen time in boys were, in decreasing order: Internet for non-study reasons (mean = 166.4 min/day, approximately 2 h and 46 min/day), console games (mean = 120.1 min/day, approximately 2 h/day), computer games (mean = 85.8 min/day, approximately 1 h and 26 min/day) and television viewing (mean = 82.1 min/day, approximately 1 h and 22 min/day).

Adolescent girls estimated their recreational screen time at 27.1 ± 19.2 h/week which is the equivalent of 3.9 h/day, and boys estimated their recreational screen time at 29.6 ± 24.4 h/week which is the equivalent of 4.2 h/day. Few adolescent girls (23.4%) and boys (24.1%) reported never or rarely having concurrent screen use. Only 5.8% of girls and 2.3% of boys adhered to the Canadian 24 h movement guideline of a maximum of 2 h/day of recreational screen time for a total of 12 (4.7%) adolescents in our sample adhering to this public recommendation. Adhering to the recommendation on recreational screen time seemed to be associated with better sleep quality in adolescents (4.48 ± 1.08 vs. 4.01 ± 0.86), but this difference was not statistically significant (*p* = 0.0694). 

There were some significant differences between adolescent girls and boys in terms of sleep quality (see Table 2). Boys had a better overall sleep quality compared to girls (*p* = 0.0364). The total mean score for sleep quality was 3.9 ± 0.8 for girls and 4.2 ± 0.9 for boys on a maximum of 6, with higher scores indicating better sleep quality. Boys also reported having less difficulty falling asleep (4.5 ± 1.5 vs. 4.1 ± 1.3, *p* = 0.0008) and reinitiating sleep after waking up at night (5.0 ± 1.1 vs. 4.6 ± 1.1, *p* = 0.0038) compared to girls. Results were similar when the subscales on falling asleep and reinitiating sleep were combined (boys: 4.8 ± 1.1 vs. girls: 4.4 ± 0.9, *p* < 0.0001).

**Figure 1:**
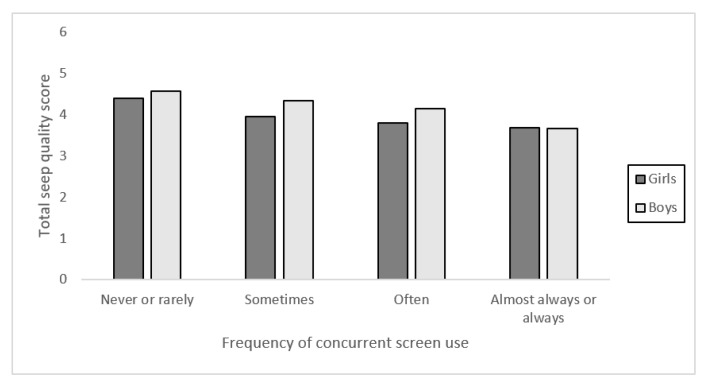
Association between frequency of concurrent screen use and sleep quality in adolescent girls and boys.

In a first model of linear regression, recreational screen time, and frequency of concurrent screen use were tested for their association with adolescents’ sleep quality (see Model 1 in Table 3). Both variables were significantly (*p* < 0.05) and negatively associated with sleep quality, and this model explained 12% of the variance in sleep quality. Adolescents’ sleep quality decreased with higher frequency of concurrent screen use (see Figure 1). In a second model, biological sex and age were added to the previous model. Being a boy (*p* = 0.0004) was significantly and positively associated with adolescents’ sleep quality while age was not (*p* = 0.6321) and this model explained 16% of the variance in sleep quality (see Model 2 in Table 3). Moderation analyses indicated that the following interaction terms were not significantly associated with adolescents’ sleep quality: (1) recreational screen time × concurrent screen use (*p* = 0.3969), (2) recreational screen time × biological sex (*p* = 0.7656), and (3) concurrent screen use × biological sex (*p* = 0.7220).

## 4. Discussion

This study confirms that adolescents had a high recreational screen time and that very few adhered to the Canadian 24 h movement guideline of a maximum of 2 h/day during the third wave (late April to mid-May 2021) of the COVID-19 pandemic in Canada. Similarly to a previous study among adolescents (12–17 years) conducted during the first wave (April 2020) of the COVID-19 pandemic in Canada [3], adolescent boys had a higher recreational screen time compared to girls in our sample. Boys in our study reported an average of 7 h and 34 min/day of total recreational screen time and girls an average of 5 h/day compared to 6.72 h/day for adolescent boys and 6.31 h/day for girls in that previous study [3]. Similarly to previous studies conducted pre-pandemic among adolescents in Canada [28] and in Norway [22], adolescent boys in our sample reported more time playing console and computer games compared to girls. Comparable to two previous studies conducted pre-pandemic among children and adolescents in Canada [26,27], a lower proportion of adolescent boys adhered to the Canadian 24 h movement guideline of a maximum of 2 h/day of recreational screen time, although this difference was not statistically significant in our study. In our sample, 5.8% of adolescent girls and 2.3% of boys adhered to the Canadian public health recommendation of a maximum of 2 h/day of recreational screen time compared to 7.9% and 5.4% during the first wave (April 2020) [3] and 16.7% and 16.4% during the second wave (April 2020) [4] of the COVID-19 pandemic in Canada.

Adolescents’ recreational screen time was negatively associated with their sleep quality. This result confirms previous studies which reported that screen time is negatively associated with adolescents’ sleep [23,24,43]. To our knowledge, this is the first study to examine this association during the COVID-19 pandemic among adolescents in Canada. Adhering to the Canadian 24 h movement guideline of a maximum of 2 h/day of recreational screen time seemed to be associated with better sleep quality, but this difference was not statistically significant. It is worth mentioning that a total of only 12 (4.7%) adolescents in our sample adhered to this public health recommendation. Age was also not significantly associated with adolescents’ sleep quality, contrary to previous studies among adolescent girls [30,32]. This may be due to the limited age range (14–18 years) in our study or because our sample also included adolescent boys.

Only 23.4% of adolescent girls and 24.1% of boys in our study reported never or rarely having concurrent screen use, suggesting that this practice is commonplace for a majority of adolescents. Concurrent screen use was negatively associated with adolescents’ sleep quality, and this association became increasingly stronger with higher frequency of concurrent screen use. Our results suggest that more frequent concurrent screen use was more detrimental to adolescents’ sleep quality during the third wave of the COVID-19 pandemic in Canada. A pre-pandemic study among adolescents (16–19 years) in Norway reported that adolescents who used many electronic devices (computer, cell phone, MP3 player, tablet, console, and television) within 1 hour of bedtime were more at risk of taking more than 60 min to fall asleep (sleep onset latency) and to lack sleep (sleep deficiency) [22]. Another pre-pandemic study among adolescent girls (11–14 years) in the United Kingdom reported that concurrent screen use after school was associated with shorter sleep duration [25]. Public health interventions aimed at improving adolescents’ sleep quality during and after the COVID-19 pandemic should prioritize adolescents with concurrent screen use.

Public health surveys should question adolescents about concurrent screen use to avoid overestimating recreational screen time if the amount of time spent using different screens is measured separately. In our study there were big differences between how many hours adolescents estimated spending weekly in recreational screen time compared to when recreational screen time was measured for each screen separately using the Screen Time-Based Sedentary Behavior Questionnaire. It is difficult to ascertain if this is truly an overestimation of adolescents’ recreational screen time or if this is due to other reasons, as there are many methodological issues in measuring accurately screen time [44]. For example, it is possible that some adolescents forgot to include certain types of screen use in their weekly estimation of recreational screen time given that this question was asked prior to the items of the Screen Time-Based Sedentary Behavior Questionnaire. Conceptualizing screen time has proven challenging; there are variations in what people consider to be screen time [44].

Being a boy was associated with better sleep quality during the third wave of the COVID-19 pandemic in Canada. Adolescent boys in our sample reported having less difficulty falling asleep and reinitiating sleep after waking up at night compared to girls. Similarly, pre-pandemic studies have reported that adolescent girls had a higher prevalence of poor sleep quality [29], reported longer wakefulness at night and lower sleep efficiency [30], and were more likely to experience insomnia symptoms compared to boys [32]. A potential explanation for these sex differences in sleep quality is that adolescent girls also reported worse sleep habits (e.g., drinking caffeine after dinner) and this was associated with increased risks of having trouble functioning (e.g., concentrating at school) the next day [29]. In our study, the sex difference in sleep quality was not explained by higher recreational screen time among adolescent girls nor by age differences between both sexes. Adolescent boys had a higher recreational screen time, and this is similar to a pre-pandemic study among adolescents (16–19 years) in Norway which reported that adolescent boys had a higher total daytime screen time compared to girls [22]. Other behavioral (e.g., caffeine consumption before bedtime) [29] and/or biological factors (e.g., hormonal fluctuations associated with the menstrual cycle for adolescent girls who reached menarche [45]) could explain the sex difference in sleep quality in our study. In fact, pubertal maturation has been associated with a higher prevalence and greater severity of insomnia symptoms in adolescent girls compared to boys [32].

Moderation analyses indicated that the association between recreational screen time and adolescents’ sleep quality was not because of concurrent screen use; they were both independently associated with sleep quality. This result suggests that both recreational screen time and concurrent screen use should be targeted by public health interventions aimed at improving adolescents’ sleep quality during and after the COVID-19 pandemic. The association between recreational screen time and adolescents’ sleep quality did not significantly vary by biological sex, even though adolescent boys had a significantly higher recreational screen time compared to girls. The association between concurrent screen use and adolescents’ sleep quality also did not significantly vary by biological sex, even though adolescent girls and boys had slightly different frequencies of concurrent screen use. The latter two results indicate that both adolescent girls and boys could benefit from public health interventions aimed at lowering their recreational screen time and concurrent screen use to improve their sleep quality, even though adolescent girls reported poorer sleep quality compared to boys in our sample.

## 5. Strengths and Limitations

This study has a few strengths and limitations. Strengths include using validated questionnaires specifically designed for adolescents to measure recreational screen time and sleep quality and verifying if adolescents had concurrent screen use. Another strength is making a distinction between screen time for study (educational) and non-study (recreational) reasons since many adolescents had remote school during the peak of COVID-19 pandemic in Canada. Only recreational screen time was measured; thereby the behavior was aligned with current public health recommendations in Canada. A limitation of our study is that we may have overestimated recreational screen time since most adolescents reported concurrent screen use and there were big differences between how many hours adolescents estimated spending weekly in recreational screen time compared to when recreational screen time was measured for each screen separately using the Screen Time-Based Sedentary Behavior Questionnaire. However, there is recent data suggesting that whether recreational screen time is passive (e.g., television viewing) or active (e.g., computer games, console games) might be more important than simply time spent using screens for child [46] and adolescent [47] health. Another limitation is that our measure of recreational screen time did not ask adolescents if they consulted screens within 1 h of bedtime while this is also detrimental to adolescents’ sleep [22]. Timing (i.e., daytime vs. evening) of screen time can have an impact on adolescents’ sleep quality and duration [48]. Finally, other behavioral variables could affect adolescents’ sleep quality as well, such as caffeine consumption before bedtime [29]. There is also data suggesting that the use of blue light-blocking glasses can mitigate the impact of evening recreational screen time on adolescents’ ability to fall asleep [49]. Future studies may want to document if passive or active recreational screen time has a more harmful effect on adolescents’ sleep quality, make a distinction between daytime and evening recreational screen time, control for other behavioral factors that can affect sleep quality, and ask adolescents whether they are using blue light-blocking glasses or the blue light filter mode on their electronic devices in the evening. Ideally, both objective and self-reported measures of adolescents’ recreational screen time and sleep quality should be used to overcome limitations associated with both types of measure [50] (e.g., reactivity for objective measures and memory bias for self-reported measures).

## 6. Conclusions

Adolescents had a high recreational screen time and very few adhered to the Canadian 24 h movement guideline of a maximum of 2 h/day during the third wave (late April to mid-May 2021) of the COVID-19 pandemic in Canada. Adolescent girls also seem at higher risk for poorer sleep quality. Public health interventions during and after the COVID-19 pandemic should suggest limiting recreational screen time and concurrent screen use and especially target adolescent girls to possibly improve adolescents’ sleep quality and promote their physical and mental health.

## Figures and Tables

**Table 1:** Characteristics of participants (*n* = 258).

Variables	*n*	%
*Age*		
● 14 years	32	12.4
● 15 years	90	34.9
● 16 years	90	34.9
● 17 years	42	16.3
● 18 years	4	1.6
*Biological sex*		
● Female	171	66.3
● Male	87	33.7
*Gender*		
● Girl	166	64.3
● Boy	85	32.9
● Neither a girl nor a boy	3	1.2
● I prefer not to answer	1	0.4
● Other	3	1.2
*School level*		
● 3rd year of high school	93	36.0
● 4th year of high school	91	35.3
● 5th year of high school	74	28.7

**Table 2:** Adolescents’ recreational screen time and sleep quality (*n* = 258).

Variables	Girls (*n* = 171)		Boys (*n* = 87)		*p*-Value
	Mean ± SD or %	Median (IQR)	Mean ± SD or %	Median (IQR)	
*Total recreational screen time (min/day)*	300.5 ± 129.3	280.7 (237.9; 365.3)	454.3 ± 197.5	451.0 (270.7; 589.1)	**<0.0001**
Internet for non-study reasons	185.1 ± 68.0	218.9 (132.9; 241.0)	166.4 ± 76.1	210.0 (90.0; 241.0)	0.0763
Television viewing	73.2 ± 62.0	57.9 (15.0; 107.1)	82.1 ± 82.0	45.0 (12.9; 150.0)	0.7785
Console games	24.7 ± 49.6	0 (0; 23.6)	120.1 ± 94.7	107.1 (15.0; 218.9)	**<0.0001**
Computer games	17.6 ± 48.4	0 (0; 10.7)	85.8 ± 100.0	23.6 (0; 197.9)	**<0.0001**
*Frequency of concurrent screen use, %*					0.2898
Never or rarely	23.4	N/A	24.1	N/A	
Sometimes	26.3	N/A	31.0	N/A	
Often	31.6	N/A	20.7	N/A	
Almost always or always	18.7	N/A	24.1	N/A	
*Adhere to the recommendation of a maximum of 2 h/day, %*	5.8	N/A	2.3	N/A	0.3479
Total sleep quality score ^†^	3.9 ± 0.8	4.0 (3.3; 4.5)	4.2 ± 0.9	4.1 (3.6; 4.9)	**0.0364**
Going to bed (3 items)	3.6 ± 1.2	3.7 (2.7; 4.3)	3.6 ± 1.4	3.7 (2.7; 4.7)	0.9527
Falling asleep (2 items)	4.1 ± 1.3	4.0 (3.0; 5.0)	4.5 ± 1.5	5.0 (3.5; 6.0)	**0.0008**
Reinitiating sleep (3 items)	4.6 ± 1.1	5.0 (4.0; 5.3)	5.0 ± 1.1	5.0 (4.7; 6.0)	**0.0038**
Returning to wakefulness (2 items)	3.3 ± 1.2	3.0 (2.5; 4.0)	3.6 ± 1.3	3.5 (2.5; 4.5)	0.0991

**Note.** SD: standard deviation; IQR: interquartile range. Numbers in bold are statistically significant (*p* < 0.05); N/A: not applicable; ^†^ Possible range of scores: 1–6, with higher scores indicating better sleep quality.

**Table 3:** Association between adolescents’ recreational screen time and sleep quality (*n* = 258).

Variables	β Coefficient (*p*-value)	
	*Model 1*	*Model 2*
Recreational screen time (min/day)	**−0.0007** **(*p* = 0.0400)**	**−0.0012** **(*p* = 0.0005)**
Frequency of concurrent screen use		
● Never or rarely	Reference	Reference
● Sometimes	**−0.3247** **(*p* = 0.0251)**	**−0.3141** **(*p* = 0.0269)**
● Often	**−0.5087** **(*p* = 0.0006)**	**−0.4147** **(*p* = 0.0048)**
● Almost always or always	**−0.6866** **(*p* < 0.0001)**	**−0.6155** **(*p* = 0.0002)**
Biological sex (being a boy)		**0.4276** **(*p* = 0.0004)**
Age (years)		−0.0256 (*p* = 0.6321)
Adjusted R^2^	0.12	0.16
Model F (*p*-value)	**8.31** **(*p* < 0.0001)**	**7.94** **(*p* < 0.0001)**

**Note.** β: standardized beta coefficient. Numbers in bold are statistically significant (*p* < 0.05).

## Data Availability

The Research Ethics Committee of the CISSS de Chaudière-Appalaches did not approve publicly sharing our dataset.
